# Highly variable sperm precedence in the stalk-eyed fly, *Teleopsis dalmanni*

**DOI:** 10.1186/1471-2148-6-53

**Published:** 2006-06-26

**Authors:** Laura S Corley, Samuel Cotton, Ellen McConnell, Tracey Chapman, Kevin Fowler, Andrew Pomiankowski

**Affiliations:** 1 Galton Laboratory, Department of Biology, University College London, Wolfson House, 4 Stephenson Way, London NW1 2HE, UK; 2 Department of Entomology, Washington State University, Pullman WA 99164-6382, USA; 3 Department of Biology, University College London, Darwin Building, Gower Street, London, WC1E 6BT, UK

## Abstract

**Background:**

When females mate with different males, competition for fertilizations occurs after insemination. Such sperm competition is usually summarized at the level of the population or species by the parameter, *P*_2_, defined as the proportion of offspring sired by the second male in double mating trials. However, considerable variation in *P*_2 _may occur within populations, and such variation limits the utility of population-wide or species *P*_2 _estimates as descriptors of sperm usage. To fully understand the causes and consequences of sperm competition requires estimates of not only mean *P*_2_, but also intra-specific variation in *P*_2_. Here we investigate within-population quantitative variation in *P*_2 _using a controlled mating experiment and microsatellite profiling of progeny in the multiply mating stalk-eyed fly, *Teleopsis dalmanni*.

**Results:**

We genotyped 381 offspring from 22 dam-sire pair families at four microsatellite loci. The mean population-wide *P*_2 _value of 0.40 was not significantly different from that expected under random sperm mixing (i.e. *P*_2 _= 0.5). However, patterns of paternity were highly variable between individual families; almost half of families displayed extreme second male biases resulting in zero or complete paternity, whereas only about one third of families had *P*_2 _values of 0.5, the remainder had significant, but moderate, paternity skew.

**Conclusion:**

Our data suggest that all modes of ejaculate competition, from extreme sperm precedence to complete sperm mixing, occur in *T. dalmanni*. Thus the population mean *P*_2 _value does not reflect the high underlying variance in familial *P*_2_. We discuss some of the potential causes and consequences of post-copulatory sexual selection in this important model species.

## Background

When females copulate with more than one partner, competition for fertilizations occurs after insemination. Such post-copulatory sexual selection can be a potent evolutionary force, as is evidenced by the numerous male behavioral, physiological, and morphological adaptations that influence sperm competition, such as mate guarding, increased copulation duration, seminal fluid induced reluctance of female re-mating, and the mechanical removal of sperm [1-3]. In addition, post-copulatory sexual selection can enhance or diminish male ornament evolution if ornament size covaries positively or negatively, respectively, with sperm competitive ability [4-6].

The most widely used metric for sperm competition that is used to infer patterns of paternity is the proportion of eggs sired by the second male in controlled double-mating trials (*P*_2_; [7]). Species or population level studies of *P*_2 _have been used extensively to describe sperm competition, particularly in insects [2] and birds [1]. However, considerable variation in *P*_2 _often occurs between populations and individuals, and intra-specific values of *P*_2 _can range from zero to one [2,5,8]. Such variation can severely limit the utility of population-wide (or species) *P*_2 _estimates as descriptors of sperm usage, because it fails to account for variation in male performance (*P*_2 _is derived from the performance of both first and second males), or aspects of female morphology and behaviour, such as sperm storage, that may differ between individual females [9]. For example, within many Lepidopteran species, some females lay eggs fertilized almost exclusively by the first male to mate, whereas others show strong second male sperm precedence ([10]; see also [11] for an example in guppies); in these instances mean *P*_2 _values do not reflect the underlying bimodal distribution of male fertilization success. So in order to fully understand the causes and consequences of sperm competition it is necessary to estimate not only mean levels of sperm precedence, but also intra-specific variation around that mean.

Numerous factors have been shown to influence intra-specific variation in *P*_2 _including male size, sexual ornamentation, mating history, reproductive organ size and diet [2,3,5]. There is also support for the hypothesis that males tailor their ejaculate in response to female factors such as size, mating status, age, fecundity and familiarity [12]. In addition, evidence is accumulating that, contrary to traditional views, ejaculates are in fact costly and cannot be produced in limitless quantities [12,13], and that male gamete availability can limit zygote formation. Under sperm-limitation, reproductive asymmetry between the sexes for variance in fertilisation success, and with it the advantage of sperm competition for available ova, will be reduced. Nonetheless, if a particular mating role is favoured (e.g. first rather than last), a male is still predicted to invest more in the favoured role [14]. Sperm-limitation phenotypes are most commonly seen in free-spawning external fertilizers at low density [15-17], although similar selective environments may be common in internal fertilisers when females receive insufficient sperm to fertilise all of their eggs [e.g. [12,18]].

Stalk-eyed flies (Diptera:Diopsidae) are increasingly important model organisms for studies of sexual selection [19-22]. They are characterised by the lateral extension of the eyes on elongate protuberances on the side of the head capsule, a trait common to both sexes in all species [19,23]. In many species the eyespan of males is greater than that of females, the result of sexual selection through female mate choice [24-29] and male-male competition [30,31].

The Malaysian stalk-eyed fly, *Teleopsis dalmanni *(formerly known as *Cyrtodiopsis dalmanni*; [32]), exhibits extreme sexual dimorphism for eyespan resulting from strong intra- and inter-sexual selection on the trait in males (*ibid*.). Females form large harems on root hairs overhanging the eroded banks of streams and males compete to control these harems [19,33]. Both sexes are highly promiscuous and mate at high frequencies (~10 and 6 times per hour in the laboratory, for males and females respectively; [34,35]). There is also some evidence that males strategically allocate more ejaculate to larger, more productive females through the production and delivery of larger spermatophores [36]. However, *T. dalmanni *spermatophores are small [37] and females store few sperm following a single mating (~140; [36]). Females are therefore sperm-limited and must copulate repeatedly to attain maximal fertility [38]. This problem is exacerbated in large, highly fecund females despite being allocated more ejaculate, as they lay a lower proportion of fertilised eggs following a single copulation in comparison with less productive females [38]. Without measures of paternity however, the value of both mate choice and multiple mating can only be inferred indirectly.

Previous research describing patterns of paternity in a congener, *T. whitei*, provides ambiguous evidence about patterns of sperm use [39,40]. Using an irradiated sterile male technique, Lorch *et al*. [39] reported that *T. whitei *exhibits first male sperm precedence. Irradiated (I) and non-irradiated (N) males were mated sequentially to females in a reciprocal mating design, and evidence for first male precedence was inferred as NI families produced offspring more frequently than IN families. Lorch *et al*. [39] also concluded that sperm mixing was important since some IN crosses produced pupae. In another study [40], female *T. whitei *were mated to a male from two different populations 24 hours apart, and offspring paternity was assigned using heritable inter-population differences in leg colour. Wilkinson and Fry [40] found no significant effect of mating order on patterns of paternity with both first and second males siring equal numbers of progeny, suggesting that sperm mixing (*P*_2 _≈ 0.5) is the predominant mode of sperm utilization. Wilkinson and Fry [40] also found some evidence for intra-specific variation in *P*_2_, as males carrying a meiotic drive chromosome produced fewer sperm than non-drive males, and hence suffered a reduction in their proportion of progeny sired.

As yet, there has been no effort to identify within-population quantitative variation in *P*_2 _in stalk-eyed flies. In this paper we investigated intra-specific, within population variation of *P*_2 _in *T. dalmanni *using controlled mating experiments and microsatellite profiling of progeny. We standardized male mating history, diet and body size in order to limit variation in male reproductive organ size, as we have shown that testes and accessory glands are reduced by mating [41] and scale positively with diet quality and body size [Rogers *et al. in prep*; Cotton & Pomiankowski *unpublished*]. We also chose sires with similar eyespan to each other to limit the potential effects on *P*_2 _of female mate choice based on male ornament size [25,27,28]. We show that even in the absence of these factors there is significant variation in sperm precedence in *T. dalmanni*, with familial *P*_2_ values ranging from zero to one. We discuss the likely causes of this variation.

## Results

A total of 662 progeny were collected from the 22 dam-sire pair families (mean total brood size ± S.D. = 30.09 ± 16.09, range 8 – 63), of which 381 were genotyped successfully at four microsatellite loci (mean genotyped brood size ± S.D. = 17.32 ± 7.92, range 5 – 35). The mean value (± S.D.) of *P*_2 _across families was 0.40 ± 0.38. This average is not significantly different from a *P*_2_ of 0.5 (*t *= 1.16, d.f. = 21, *P *= 0.26).

**Figure 1:**
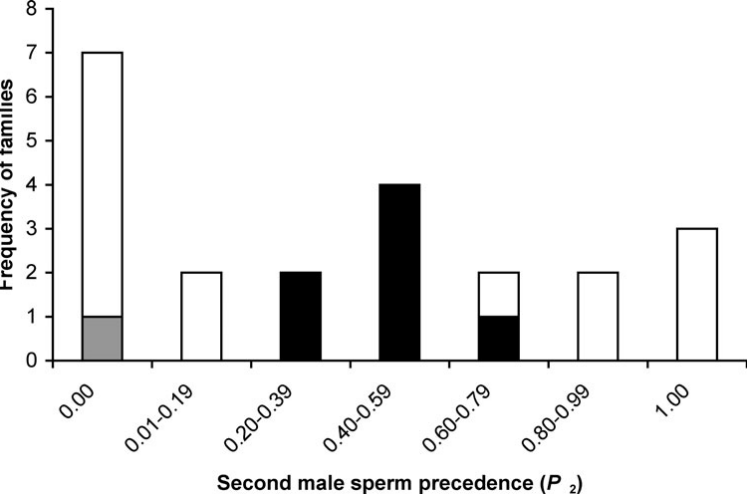
The distribution of second male sperm precedence (*P*_2_) among 22 dam-sire pair families in the stalk-eyed fly, *Teleopsis dalmanni*. Open bars depict the frequency of families with *P*_2 _significantly different from 0.5 (using 2-tailed binomial tests); black bars denote those with *P*_2 _not significantly different from 0.5. The grey bar denotes the sperm precedence of a single family where the observed *P*_2 _of zero was not significantly different from *P*_2 _= 0.5. However, the brood size of this family was small (*n *= 5), so this result should be treated with caution.

We found significant variation between the different families in terms of their *P*_2 _(*G*_*H *_= 292.09, d.f. = 21, *P *< 0.001). The pattern is approximately tri-modal, with *P*_2 _peaks at approximately 0, 0.5 and 1 (Figure 1). Ten families (45%) exhibited extreme second male biases resulting in zero or complete paternity (*n *= 1 and 3, respectively; Fig. 1). These results were unlikely to be due entirely to one of the males being infertile in all three of their matings, as the incidence of *P*_2 _= 0 or 1 was greater than that expected given the level of male infertility (over three matings) in the population (22.7% *vs*. 12.5%, see Methods § *χ*^2 ^= 9.14, d.f. = 1, *P *= 0.002). Moreover, heterogeneity of *P*_2 _was not entirely attributable to these families, as a replicated goodness-of-fit test using families with *P*_2 _≠ 0 or 1 was still significant (*G*_*H *_= 60.55, d.f. = 11, *P* < 0.001). Eight families (36%) displayed patterns of random paternity (i.e. their *P*_2 _was not significantly different from 0.5; Figure 1). The remainder had significant moderate (but not extreme) paternity skew, reflecting both first and second male sperm precedence (Figure 1).

First and second males did not differ consistently from each other for eyespan (*t *= 1.21, d.f. = 21, *P *= 0.24) or thorax length (*t *= 0.00, d.f. = 21, *P *= 1.00). Male morphology did not explain variation in *P*_2 _(first male eyespan *r *= -0.28, d.f. = 21, *P *= 0.20, second male eyespan *r *= -0.16, d.f. = 21, *P *= 0.47, first male thorax *r *= 0.10, d.f. = 21, *P *= 0.65, second male thorax *r *= -0.11, d.f. = 21, *P *= 0.62). Relative male eyespan did not explain variation in *P*_2 _(thorax included in model: first male relative eyespan *F *= 2.91, d.f. 1,19, *P *= 0.10, second male relative eyespan *F *= 0.27, d.f. = 1,19, *P *= 0.61), and neither did the difference in morphology between first and second males explain variation in *P*_2 _(eyespan difference *r *= -0.09, d.f. = 21, *P *= 0.68, thorax difference *r *= 0.16, d.f. = 21, *P *= 0.47). Female body size (thorax length) had no significant effect on *P*_2 _(*r *= -0.10, d.f. = 19, *P *= 0.66), and neither did female eyespan (*r *= -0.37, d.f. = 19, *P *= 0.10), although the latter did reveal a trend for females with larger eyespans to produce broods with fewer offspring sired by the second male.

Our data are unlikely to be affected by biased sampling of progeny within broods (with respect to male order) or variable brood sizes, as there was no correlation between the proportion of the brood sampled and *P*_2 _(*r *= 0.15, d.f. = 21, *P *= 0.51), or between total or genotyped brood size and *P*_2 _(total brood size *r *= -0.03, d.f. = 21, *P *= 0.89; genotyped brood size *r *= 0.03, d.f. = 21, *P *= 0.91).

## Discussion

Using controlled mating experiments, assigning paternity using microsatellite markers, we demonstrated that sperm usage is highly variable in the stalk-eyed fly *T. dalmanni *(Figure 1). Almost half of the dam-sire pair families genotyped had extreme first or second male paternity biases (*P*_2 _= 0 or 1, respectively). Sperm precedence was not the only mode of sperm utilization, since the null model of random sperm mixing (i.e. *P*_2 _= 0.5) explained patterns of paternity in over one third of families. Furthermore, a number of families displayed first or second male sperm precedence in conjunction with varying degrees of sperm mixing, resulting in moderate, but significant, paternity biases (i.e. 0 <*P*_2 _< 0.5 and 0.5 <*P*_2 _< 1, respectively). Thus all modes of sperm usage were found in *T. dalmanni*, and *P*_2 _exhibited a tri-modal distribution (Figure 1).

What are the causes of such variation in paternity? We can exclude some factors which our experimental design deliberately set out to minimise. It is unlikely that our data can be explained by (cryptic) female choice for male ornaments or other aspects of external morphology, a phenomenon seen in numerous other species [1-3,5], since first and second males did not differ in eyespan, the male sexual trait, or body size. As male eyespan is an accurate indicator of accessory gland and testis size in *T. dalmanni *[Rogers *et al. in prep*; Cotton & Pomiankowski *unpublished*], differences in reproductive organ size are also unlikely to be explanatory variables, even though we did not explicitly measure these traits.

Male mating history is often associated with changes in male investment in current mating attempts, with concomitant effects on patterns of paternity [12,13]. However, all males were virgins at the start of the experiment and performed equal numbers of copulations, so variation in mating experience did not differ between first and second males, and hence is not an explanation of our data. In addition, all males were maintained on a high quality diet, so variation in environmental conditions was minimised between pairs of sires. These factors (when variable) may well be important in determining stalk-eyed fly paternity and deserve further investigation, but they cannot explain the results reported in the current study.

In contrast, female mating history was not constant across sires (only the first male mated with a virgin). In promiscuous species, such as *T. dalmanni*, it is likely to be advantageous for a male to mate with a virgin female as they only risk defence, not offence, sperm competition, and theory suggests that males should on average invest more in their favoured mating role (i.e. first *vs*. second) when females are sperm limited [14]. However, this hypothesis can be refuted, as the average value of *P*_2 _was not significantly different from 0.5.

Another possible explanation is that male infertility explains the variation in *P*_2 _seen in our study. A recent simulation study showed that tri-modal distributions can be generated by male infertility in the range 10–30%, given that relative fertilization success is randomly allocated to each male [18]. Whilst this simulation study is not directly applicable to our experiment, it does point out that male infertility alone can generate a high frequency of *P*_2 _= 0 or 1. Even though infertility is quite common in *T. dalmanni *(estimated as 12.5% for males mated three times [38]), it is not high enough to explain the incidence of families with extreme paternity bias in our study (almost half had *P*_2 _= 0 or 1, which would require a rate of infertility equal to 22.7%). However, infertility seems a good explanation of a major part of the frequency of *P*_2 _= 0 or 1. In addition, partial infertility due to insemination failure of one or two of the three matings made by each male could account for a large fraction of the variation observed between 0 <*P*_2 _< 1. Another hypothesis that can generate a tri-modal distribution is "sloppy" sperm mixing. Harvey and Parker [42] report that when sperm from each male's ejaculate tend to clump together and females only use a small proportion of the sperm they receive to fertilise their eggs, high variance and multi-modal *P*_2 _distributions will be common. There is no information about sperm usage in stalk-eyed flies, so it is difficult to evaluate this idea. Further experimental work is needed to evaluate the relative importance of these hypotheses in explaining our data.

It is possible that males varied their ejaculate expenditure in relation to the reproductive value of females, in conjunction with their perceived mating status, as is observed frequently in other species [reviewed in [12]]. Since large eyespan females have higher fecundity ([36]; Cotton & Pomiankowski *unpublished*) and are also more sperm limited [36], first males are predicted to invest relatively more in large eyespan, virgin females. We found some support for this in the form of a (albeit non-significant) negative correlation between female eyespan and the *P*_2 _of a brood; males mating first with a large eyespan female showed a tendency to sire more offspring that the subsequent male. Another possibility is that males varied in aspects of external morphology or behaviour, independent of male body and ornament size, which affected male fertility. For example, males may have differed in the size or shape of their intromittant organs [43] and/or their copulatory courtship behaviour and this may have altered their ability in sperm competition, as has been reported in other insects [3,44,45].

The presence of such extreme variation in paternity means that post-copulatory sexual selection will have a profound influence on the effects of pre-mating biases, and may partially explain the high mating rate seen in *T. dalmanni*. Female *T. dalmanni *mate frequently with both the same and different males, and prefer to mate most often with large eyespan males [25,27,28]. Repeated mating is beneficial for females since it helps to alleviate sperm limitation and increases the number of fertile eggs [36,38]. A high mating rate is also beneficial for males as it increases the number of females inseminated in a harem, and also the sperm load within multiply mated individual females. In the context of the current study, the latter may be particularly important given inter-male competition and the high incidence of sperm mixing.

Variable *P*_2 _means that pre-copulatory mate choice is somewhat paradoxical both in terms of direct fertility benefits and indirect "good genes" benefits. Superficially it appears that the male that a female prefers to mate with is not necessarily the male that will sire her offspring. This paradox can be resolved however, with the observation that preferred males with large ornaments also have larger testes and accessory glands [Rogers *et al. in prep*.; Cotton & Pomiankowski *unpublished*]. Accessory gland size covaries both phenotypically [41,46] and genetically [47] with male mating frequency, and number of sperm stored in a female's spermathecae correlates positively with the testis size of her mate [48]. Therefore, the advantages of choice (both direct and indirect; Rogers *et al. in prep*; [49]) for exaggerated ornaments might be maintained because large eyespan males can minimise the uncertainty arising from variable paternity by mating more frequently and out numbering the sperm of other males. However, further experiments are required to specifically test these hypotheses.

## Conclusion

We have shown that the pattern of sperm usage is highly variable in the stalk-eyed fly, *T. dalmanni*. This greatly limits the utility of population-based estimates of *P*_2 _as descriptors of sperm usage, and suggests that sperm precedence should be viewed as context-specific, rather than a general, constant, metric in stalk-eyed flies. The unexplained variance in male fertilization success found by this study requires further investigation in order to evaluate potential causes and consequences.

## Methods

### Study animal

The laboratory population of *T. dalmanni *used in this experiment was derived from wild caught flies collected from Malaysia in 1993 by AP. Flies have been maintained in cage culture at 25°C on a 12:12 light/dark cycle, at high population size (>200 individuals) to minimize inbreeding. This population does not carry *X*-linked meiotic drive, which is found in natural populations of *T. dalmanni *[50]. The light regime included a 15-min 'dawn' period in which the culture room was illuminated by a single 60 W light bulb. All observations of mating behaviour commenced at the start of the dawn period. Experimental flies were collected as eggs and reared under low larval density [51]. Emerging adults were segregated by sex and measured for eyespan and thorax length [51] to an accuracy of 0.01 mm using a monocular microscope and the image analysis program NIH Image (Version 1.55; National Institute of Health, Bethesda, MD, USA). Flies were measured "blind" by a single person (EM). All individuals used in this experiment were sexually mature (6–8 weeks post eclosion) at the start of each experiment and fed high quality food (puréed corn) *ad libitum*.

### Mating design

Individual males were anaesthetized on ice and their left middle tibia was removed 2 weeks prior to the mating trials, to yield DNA for pre-screening microsatellite genotype (*n *= 96). Pilot mating trials showed that tibia removal had no effect on the ability of males to mount and mate with females. Males were genotyped at each of four microsatellite loci ([52], see below for details), and pairs were formed that maximised allelic differences between them, to give a total of *n *= 30 pairs. All of these male pairs differed by at least one allele for at least two loci.

In the mating trials, a single virgin female was placed in a container with one randomly chosen male (male 1) from a pair of males, at artificial dawn (lights on). The female and male were observed until three matings of 30 seconds or longer had occurred (spermatophore transfer usually occurs after copulations of 30 seconds). The male was then removed. At dawn on the second day, the second male (male 2) from the pair was placed with the female, and observed until three matings of 30 seconds or longer had occurred. The second male was then removed. Three matings per male were used to reduce the incidence of complete infertility [38].

Mated females were provided with food and water *ad libitum *and eggs were collected every two days for the following 10 days. All females were frozen on the last day and their abdomens were used to obtain DNA for genotyping. To maximise survival, eggs were allowed to develop into pupae at low larval density [51] at which time they were sacrificed for genotype analysis. One cross was excluded from the analysis because one of the males failed to achieve 3 copulations within 2 hours. A further 7 crosses were excluded from the analysis because there was allele sharing between the female and one (or both) males which precluded assignment of offspring paternity. This left 22 families for which we could estimate *P*_2_.

### Microsatellite genotyping

DNA isolation from male middle tibia, female abdomens, and whole pupae was conducted with slight modification to the protocol of Holehouse *et al*. [53]. Briefly, the tissue was ground in 45 of μl Tris-EDTA buffer (pH 8.0) with 10 μl of 20 mg/ml proteinase K (Sigma Aldrich) and then incubated for 30 minutes at 55°C and 10 minutes at 100°C. Multiplex PCR was performed according to Wright *et al*. [52] using a 1:10 dilution of extracted DNA and 10 μM primers for ms-039, ms-090, ms-301A, and ms-402 (Table 1). PCR conditions were as follows: denaturation at 94°C for 2 minutes followed by 30 cycles of 94°C for 30 sec, 55°C for 30 sec, 72°C for 65 sec, followed by 72°C for 10 minutes. Fluorescently labeled PCR products were separated on a 3100 DNA Analyzer (Applied Biosystems) and analyzed with Genescan 3.1.2 software (Applied Biosystems). Alleles from all 4 loci were scored for all individuals in the study.

**Table 1:** Microsatellite loci used in this study; repeat motif, allele size range in base pairs, and nucleotide sequence all from Wright *et al*. [52].

**Locus**	**Repeat Motif**	**Product Size (bp)**	**Primer Sequence**
ms-039	[AC]2AA[AC]4GCW[CA]3A[AC]7AT[AC]1TC[AC]2	147	F:FAM-AATCACAACGCTAACGAGTCA
			R:ATGCTTCAACGCTTACCTACC
ms-090	[GT]11GA[GT]3GG[GT]4AT[GT]1	197	F:FAM-TCTTGCCTTTGCCACACTAA
			R:TGGGAAATGTGAGTTTACTTAAACAGT
ms-301A	[AC]8AT[AC]3TC[AC]2	138	F:HEX-TTCAGCACTAAATGCAGCAGA
			R:GCACTTAACATGCGATGAGG
ms-402A	1. [CAA]2ATA[CAA]8CAG[CAA]2	205	F:HEX-CCAAATGGGCCACATTATTC
	2. [AC]8		R:AGGAAAGTGGATGCATTCGT

### Statistical analysis

We tested for consistent differences in male size between first and second males in the mating trials using paired *t*-tests for absolute eyespan and thorax length. Second male sperm precedence (*P*_2_) was calculated as the proportion of offspring from a brood sired by the second male to have mated with the female (*P*_2 _= *n*_2_/(*n*_1_+*n*_2_), where *n*_*i *_equals the number of offspring from male *i*). We tested for heterogeneity in *P*_2 _among families using the *G*_*H *_statistic from a replicated goodness-of-fit test, compared against a *χ*^2^-distribution with number of families minus 1 degrees of freedom ([54], p. 715). The numbers of offspring sired by each male, rather than proportions, were used in the derivation of *G*_*H*_. Each family was also tested for its adherence to a null-hypothesis of *P*_2 _= 0.5 (i.e. paternity distributed at random) using 2-tailed binomial tests ([55], p. 533). Extreme values of *P*_2 _(1 or 0) may have arisen from functional infertility of the first or second male, respectively. We therefore asked whether the incidence of these events were significantly greater than that expected given the standing level of functional infertility in the population for males mated 3 times (estimated as 12.5% by Baker *et al*. [38]). We note that this test is conservative as Baker *et al*. [38] defined males as infertile if their hatching success was < 10%. Correlates of sperm precedence were performed using arcsine-transformed data (arcsin P2
 MathType@MTEF@5@5@+=feaafiart1ev1aaatCvAUfKttLearuWrP9MDH5MBPbIqV92AaeXatLxBI9gBaebbnrfifHhDYfgasaacH8akY=wiFfYdH8Gipec8Eeeu0xXdbba9frFj0=OqFfea0dXdd9vqai=hGuQ8kuc9pgc9s8qqaq=dirpe0xb9q8qiLsFr0=vr0=vr0dc8meaabaqaciaacaGaaeqabaqabeGadaaakeaadaGcaaqaaiabdcfaqnaaBaaaleaacqaIYaGmaeqaaaqabaaaaa@2F03@). Statistical analyses were performed using JMP software (version 5, SAS Institute, Gary, NC, USA).

## Authors' contributions

LSC conceived the study, contributed to the design and execution of the experiment, and helped to draft the manuscript. SC conducted the statistical analysis and drafted the manuscript. EM performed the experiment and carried out the molecular genetic analyses. TC, KF and AP jointly conceived the study with LSC, participated in the design and coordination, and helped to draft the manuscript. All authors have read and approved the final manuscript.
